# Does Conflictual Dialogue Improve a Story?

**DOI:** 10.5964/ejop.17499

**Published:** 2026-02-27

**Authors:** John W. Berks, Matt N. Williams

**Affiliations:** 1 Psychological Medicine, University of Auckland, Auckland, New Zealand; 2 School of Psychology, Massey University, Auckland, New Zealand; Università Cattolica del Sacro Cuore, Milan, Italy

**Keywords:** conflict, stories, narrative, drama, dialogue

## Abstract

According to creative writing experts, adding conflict to dialogue improves story quality and audience reaction to that story. This idea draws from the widely held theory that conflict is vital for dramatic stories. Yet despite conflict’s seeming centrality, experimental research into its effects is limited. We take a small step in addressing this shortfall, by examining whether one type of conflict — adversarial dialogue — improves story quality, and audience response. In a pilot, and two similar studies, we manipulated stories to create different levels of conflictual dialogue, in repeated measures experiments. Forty-seven participants in the first study and 194 in the second, read stories with different levels of conflict, and then rated them on a series of measures (the Perceived Quality Index in the first experiment, the Audience Response Scale, with supplementary questions on boredom and story quality in the second). While the conflict manipulation was successful, it produced no significant difference in these outcome measures in the two experiments. These results do not support the study hypothesis that manipulating isolated adversarial dialogue has a positive effect on stories but would be compatible with alternative theories of conflict in stories.

Stories are ubiquitous. Everywhere, from the media to ordinary conversations, people relate stories and respond to stories. In the construction of these stories, conflict is widely theorized to be important. Yet experimental research into story conflict is sparse (Ware, 2011), and we have identified only three previous experiments which manipulate story conflict and then measure people’s reaction — Diener and Woody (1981), Brewer and Lichtenstein (1981), and Jose (1988) — and only in the former, is story conflict a focus of the research. Therefore, it is timely to return to this topic. This paper presents a pilot study and two experiments that manipulate one type of conflict in stories, adversarial dialogue, and then measure audience reaction. While aimed particularly at psychology researchers and writers, this research is intended for anyone who is interested in stories and people’s response to them.

According to creative writing experts, dialogue is a crucial element of writing, and adding conflict to dialogue improves story quality and audience reaction to a story (Axelrod, 2013; Bell, 2014; Frey, 2010; Hough, 2015; McKee, 2016; Stein, 1995).

While this practical advice is not based on empirical research, it does draw from broader theorizing on the necessity of conflict for a good dramatic story, an idea expressed across a range of disciplines (Bruner, 1986; Brunetière, 1914; Egri, 2009; Freytag, 1896; Gellrich, 1984; McKee, 1999; Vorderer & Knobloch, 2000; Ware et al., 2012). Although this emphasis on story conflict has been identified across cultures (Hogan, 1997), the modern Western consensus that conflict is necessary for a good story probably derives from Georg Hegel (Gellrich, 1984; Lawson, 1949). Hegel’s idea was that the clash, between the will of the story protagonist and opposing forces, propelled a story forward through a series of *tragic collisions*, culminating in the resolution of these forces (Hegel, 1998; Lawson, 1949).

Other theorists followed Hegel in emphasizing a protagonist with a goal, and antagonists and obstacles in opposition to that goal (Bruner, 1986; Egri, 2009; McKee, 1999; Stein, 1995; Vorderer & Knobloch, 2000). This goal could be driven by different motivations (Hahn et al., 2017; Lewis & Mitchell, 2014); and the obstacles could be internal, or external (Brunetière, 1914; Hahn et al., 2017; Lawson, 1949; McKee, 2016). Some recommended that conflict should build to a high point and then resolve (Egri, 2009; Freytag, 1896). Conflict can be intensified by closely matched opposing sides, “closeness” of participants (geographically, and in their relationship to each other), and high stakes (Ware et al., 2012). Most theorists also followed Hegel in stressing the importance of careful plotting to create the tragic collision, producing what is variously described as drama (Brunetière, 1914), action (Egri, 2009), or conflict (McKee, 1999).

In this model, which we will call the traditional theory, we discern three elements used to generate conflict: a character with a goal and obstacles to that goal, a plot which brings the goal and obstacles together, and the depiction of the ensuing drama. In this article we combine the first two elements under the label *structural conflict*, while the third will be referred to as *dramatic conflict*, an example of which would be adversarial dialogue.

Is structural conflict therefore necessary for a positive reaction to dramatic conflict? A close link between structural and dramatic conflict is not always overt in the creative writing literature, and while it is rare for a writing expert to advise indiscriminately adding dramatic conflict to improve a flat scene, in practice writers do employ this strategy. The detective novelist, Raymond Chandler, famously wrote “…the demand was for constant action; if you stopped to think you were lost. When in doubt have a man come through a door with a gun in his hand. This could get to be pretty silly, but somehow it didn’t seem to matter.” (Chandler, 1946). This implies a simpler explanation, not directly expressed in the literature — that dramatic conflict, unconnected to structural conflict, can arouse positive emotional/cognitive responses in an audience. Presumably these positive responses would either occur through improved story quality; or through having a direct effect on an audience — an idea similar to the theory that violence in media has a direct effect on an audiences enjoyment (Hoffner & Levine, 2005). We will call this model the dramatic theory of conflict.

Why might conflict improve story quality or audience reaction?

Story quality has a number of components, including plot, theme, and characterization. Hegel’s idea that the plot is driven forward by dramatic conflict is echoed by creative writers (Lawson, 1949; Swain & Swain, 1988). Egri (2009), thought the theme of a story was created by structural conflict, while dramatic conflict illustrated this theme. For other theorists, the benefit of dramatic conflict was in revealing character (Frey, 1987; Lawson, 1949; McKee, 1999). How these improvements in story quality lead to a positive audience response is not explicit in this literature.

For literature that does make a connection between conflict and audience reaction, we turn to psychology, and theories we will group under the label *the psychological theory of conflict.*

The affective disposition theory (ADT) of drama (Zillman & Cantor, 1977) posits that people decide which characters they like or dislike through a character’s good or bad actions (Raney, 2002). Enjoyment of the drama then depends on a successful resolution of the story for liked characters or an unsuccessful resolution for the disliked characters. In drama, these successes and failures typically arise from the resolution of external conflict (Raney, 2013). Additionally, anticipation of these good or bad resolutions of conflict, has been theorized to cause suspense, and this suspense leads to enjoyment (Vorderer & Knobloch, 2000).

To explain a positive audience response to internal conflict, or stories with unhappy endings for liked characters, we draw on appreciation theories (Oliver & Bartsch, 2010; Tamborini, 2013; Vorderer et al., 2004). Appreciation theories do not focus on story conflict, but rather on the resulting conflict in the audience. *Cognitive conflict* and *moral conflict* arise in an audience when they struggle to make sense of stories featuring justice violations or moral dilemmas, and these conflict lead to an appreciation response (Bartsch & Hartmann, 2017; Grizzard et al., 2021; Lewis et al., 2014; Tamborini, 2013).

However, while story conflict appears necessary for these audience responses, whether manipulating this story conflict leads to changes in enjoyment, appreciation or suspense is not a focus of the psychological theory of conflict.

These three theories of story conflict are summarized in Table 1.

**Table 1:** Theories of How Conflict Improves Story Quality and Audience Response

Theory	Summary
Psychological	Manipulation of the valence of character and/or the valence of the resolution of a dramatic conflict (two independent variables), changes audience responses (dependent variables). Conflict is a potential moderating variables to this relationship (as is suspense). If this audience response is appreciation (dependent variable), audience internal conflict is a mediating variable to this relationship.
Traditional	Structural conflict (independent variable) heightens dramatic conflict (mediating variable). Heightened dramatic conflict improves story quality (mediating variable), which increases positive audience responses (dependent variables).
Dramatic	Dramatic conflict (independent variable) increases positive audience responses (dependent variables), possibly via improving story quality (mediating variable).

Moving from theory to evidence, there has been no empirical research investigating whether adversarial dialogue is superior. However, there are studies involving manipulation of conflict which show increased liking both of stories with high “emotional conflict” (Diener & Woody, 1981), high stakes goals (Brewer & Lichtenstein, 1981), and stories with significant obstacles to goal achievement (Jose, 1988). In these studies, structural and dramatic conflict are not overtly differentiated. However, the studies of Brewer and Lichtenstein, and Jose give enough detail to suggest the former manipulates structural conflict, while the latter manipulates both structural and dramatic conflict.

More research examines the mechanisms of positive audience response to stories containing unmanipulated conflict.

Conflict is used in story research to create suspense (Comisky & Bryant, 1982; Doust, 2015; Gerrig & Bernardo, 1994; Madrigal et al., 2011; Vorderer & Knobloch, 2000; Zillmann et al., 1975), and research show that suspense leads to story enjoyment (Brewer & Lichtenstein, 1981; Madrigal et al., 2011), however, we lack experimental evidence showing the whole pathway: increasing conflict increases suspense, in turn increasing story enjoyment. Similarly, though research on the ADT of drama has shown that the resolution of a story conflict in favour of a liked character leads to enjoyment (Grizzard et al., 2023; Hoffner & Cantor, 1991; Raney & Bryant, 2002; Zillmann, 2000; Zillman & Cantor, 1977), and appreciation research has shown that a mixed valence story resolution for a liked character leads to appreciation (Bennie et al., 2020; Grizzard et al., 2021; Knop-Huelss et al., 2020; Lewis et al., 2014), none of this research involved manipulation of conflict.

So, in this literature there are a number of gaps, including experimental research manipulating isolated structural, and dramatic conflict. The latter variable is theoretically significant, because dramatic conflict is central to the dramatic theory, and part of the causal pathway of the traditional theory of conflict. As a moderating variable of unknown strength, dramatic conflict’s importance is less certain in the psychological theory.

In addition to these theoretical issues, the experiments presented here also have a practical purpose: To investigate how to improve dialogue — an important component of a well written story (Axelrod, 2013; Bell, 2014; Frey, 2010; Hough, 2015; McKee, 2016; Stein, 1995).

## Creating Adversarial/Conflictual Dialogue

Adversarial dialogue is one place where dramatic conflict could be isolated from structural conflict. Not every passage of dialogue is a manifestation of the clash between story goal and obstacle. Dialogue is used for other purposes, such as exposition or to reveal character (Hough, 2015), where the dramatic conflict is created through providing additional motivation for characters to be in conflict, such as the desire for social precedence, or having different attitudes to life (Hahn et al., 2017; Lewis & Mitchell, 2014; Stein, 2014).

Complementary to this motivation, dramatic conflict is realized through dialogue content, how it is delivered, and accompanying body language (Kempton, 2004; Stein, 2014). These conflict behaviours are sometimes listed: “Your characters can: threaten, tease, argue, wheedle, cajole, insist, taunt, demand, interrupt, lie” (Patterson, 2013).

## Hypotheses: Experiment 1 and Pilot

To help address the gap in knowledge about dramatic conflict and its effects on story quality and audience reaction, we tested the following hypotheses.

*Hypothesis 1 (H1)*: High levels of dramatic conflict in story dialogue will result in higher story quality ratings than low levels of dramatic conflict.
*Hypothesis 2 (H2)*: High levels of dramatic conflict in story dialogue will result in more positive audience response ratings than low levels.

## Pilot Study

A pilot study tested the technical aspects of the study and assessed the practicality of the manipulation. This pilot, in a design similar to Experiment 1 (see Method section, Experiment 1 for details), had twenty-three participants rate stories in which the dialogue had been manipulated into different levels of conflict. An important difference was the level of conflict was manipulated into three levels rather than two in subsequent experiments. There was a base, low conflict condition. Adding conflict behaviours to create the medium conflict condition, led to an insignificant, small to medium increase in readers’ perceived conflict ratings (see Table 2). However, adding both conflict behaviours and motivation for conflict to create the high conflict condition, led to a large and significant effect when compared to the medium conflict condition, and a highly significant, large to very large effect, compared with low conflict condition (see Electronic Supplementary Material 1, ESM 1 at Berks, 2026).

**Table 2:** Perceived Conflict in the Pilot

Conflict levels compared	MD	95% CI	*t*-value	*p*-value	Cohen’s *d*
High & Med	0.87	[-1.53, -0.21]	2.75	.01	0.57
High & Low	1.35	[-1.93, -0.77]	4.84	<.001	1.01
Med & Low	0.48	[-1.19, 0.23]	1.39	.18	0.29

This suggested that manipulating the motivation for dramatic conflict was the most important manipulation, but the combination of both manipulations was strongest. Therefore, in Experiment 1, the design was simplified by combining the two manipulations, to create two levels of conflict: low (no manipulation) and high (double manipulation).

While there were no further significant results, the mean difference scores for the audience response variables all showed higher scores in the high conflict versus low conflict story versions (see Tables ESM 1.1 and 1.2 at Berks, 2026). This was suggestive that a study with greater power would show a significant increase in audience response scores with high conflict.

## Method: Experiment 1

### Participants

#### Recruitment

Recruitment was via the online website Prolific. To avoid comprehension problems, participants were recruited with English fluency using Prolific’s prescreening. Sixty-nine participants entered the study. After exclusions, 47 participants remained (see ESM 2 at Berks, 2026).

The exclusions included six participants in Condition 7 (see Table 3), who got no second story, due to an error in the pseudo-randomization, and so were excluded. These latter exclusions are possibly best characterised as data missing at random (Heitjan, 1994). To assess whether this loss of Condition 7 had a meaningful effect on the analysis, a multilevel model was added (see ESM 1 at Berks, 2026).

**Table 3:** Twelve Participant Condition Combinations, Randomly Assigned

	Condition Combinations											
Presentation Order	1	2	3	4	5	6	7	8	9	10	11	12
1^st^ Story	FH	FH	FL	FL	RH	RH	RL	RL	CH	CH	CL	CL
2^nd^ Story	RL	CL	RH	CH	FL	CL	FH	CH	FL	RL	FH	RH

*Note.* F = Fantasy, R = Romance, C = Crime; L = low conflict, H = high conflict

#### Sample Size/Power Analysis

The study had a target sample size of 59 based on the power analysis.

There is no easy comparison in the literature for this research, and data from the pilot was inconsistent, but suggested a small to medium effect size (Cohen, 1988) for both story quality measured with the Perceived Story Quality Index (PSQI), and Entertainment measured by a question in the PSQI (Baron & Bluck, 2011). To detect a difference between the ‘low’ and ‘high’ conditions, an *a priori* sample size analysis using G*Power 3.1 (Faul et al., 2007), using a two-tailed paired *t*-test, and a Cohen’s *d* of 0.35 (alpha = .05; desired power = 80%), suggested a minimum sample of 59 was required. Given a loss of data from exclusions, the recruitment target was set at 70.

### Design

The design was a repeated measures experiment with two levels of the independent variable, dramatic conflict, low and high; and two dependent variables, story quality and Entertainment.

### Procedure

On the Qualtrics online platform, participants were randomized into one of twelve conditions. In each condition they read two different stories, one featuring a low conflict dialogue passage, and one a high conflict passage. After reading the dialogue portion of each story, the participants rated the story’s quality and Entertainment and answered a manipulation check.

The twelve conditions were created as follows. To avoid boredom and unblinding, each participant was given two different stories, rather than ask them to read the same story twice. To increase the generalizability of the study, these two stories were drawn from a pool of three stories — a fantasy, a romance and a crime story. Each of these three stories had a high and a low conflict version. This produced the twelve possible condition combinations, as shown in Table 3.

Qualtrics presented these conditions in a pseudo-random pattern that ensured that there was an even distribution.

### Materials

#### Stories

Because of the study’s idiosyncratic story requirements, new stories were created. Although this study did not focus on testing effects of story genres, Story 1 was written as a fantasy story, Story 2 a romantic comedy, and Story 3 as a crime story (see ESM 3 at Berks, 2026). Variation between stories goes beyond genre, and so are referred to as genre/story type.

##### Creating Conflict

Because the tools for manipulating conflict, described in the introduction, can be varied, and combined in many ways (Hahn et al., 2017; Lewis & Mitchell, 2014; Patterson, 2013; Stein, 2014; Ware et al., 2012), we developed guidelines:

- Techniques outlined under Adversarial dialogue, should be used to increase dramatic conflict.
- Dramatic conflict should be increased as much as possible, from the low to high conflict versions.
- Other than dramatic conflict, the two versions of each story should be kept as similar as possible.

The latter guideline aimed to minimize the effect of extraneous variables and avoid manipulation of structural conflict. Therefore, we decided not to manipulate either conflict intensifiers, nor the story goal and the obstacles to that goal. Instead, we provided a motivation for conflict (see Table 4) unconnected with the main story goal and showed the resulting dramatic conflict through conflict behaviours (see ESM 4 at Berks, 2026). No attempt was made to manipulate character liking, nor the valence of conflict resolution.

**Table 4:** Key Elements in the Introduction From the Three Stories

Story	Characters	Story goal	Intensifiers	Motivation for conflict
Fantasy
	Lo min Shaggor	Lo min wishes to fly.	Evenly matched characters. Meet face-to-face. Shaggor is engaged to Lo min’s sister.	The characters dislike each other.
Romance
	Wendy Shay	Wendy wants to catch a man’s attention.	Evenly matched characters. Meet face-to-face. Shay is Wendy’s sister.	The characters have different attitudes to life.
Crime
	Ali Zach	Ali wishes to prove her client’s innocence.	Evenly matched characters. Meet face-to-face.	The characters have different attitudes to life.

Each story was divided into three parts.

In the first, a short Introduction identified the characters, the protagonist’s goal, conflict intensifiers, and in the high dramatic conflict version, the motivation for the character conflict. These key elements of each story are shown in Table 4.

In the main part of the story, the dialogue occurs. To create the high conflict version, the neutral dialogue in the low conflict version was revised line by line to add conflictual content and accompanying behaviour. For example, here is the low conflict version of an exchange between Lo min and Shaggor, as they discuss scaling a giant pillar to meet the wizard Baal-pteor:

“Remember, Baal-pteor cannot be harmed by weapons wielded by human hand,” said Lo min. “And it is a tricky climb”. Once again, Shaggor studied the pillar, then with evident reluctance, abandoned the sword.

Compare the high conflict version:

“You forget, Baal-pteor cannot be harmed by weapons wielded by human hand,” said Lo min. “But if having the sword lessens your fear…”. Shaggor glared at Lo min, but he abandoned the sword.

The neutral content “Remember” is replaced by the implied criticism of “You forget”, the neutral advice about the climb is replaced by insinuations of cowardice, and the neutral body language of studying the pillar is replaced by a glare.

The third part resolved the story. As this did not manipulate the independent variable, there was only one version of the third part.

##### Measures

In studying the effects of dramatic conflict we wanted a measure of story quality, and broad measure of audience response, such as Entertainment (Klimmt, 2011; Vorderer, 2001), or a range of more specific responses, potentially including boredom, interest, enjoyment, appreciation, tension, and suspense.

The PSQI of Baron and Bluck (2011) was used for Experiment 1 because it gives an overall rating of story quality, and contains multiple dimensions, including an item for Entertainment: “To what extent is this story entertaining?” The PSQI has six items, of which two are negatively worded and reversed for scoring. Responses are made on a 5-point Likert-type scale with scores ranging from 1 to 5. Total scores for the PSQI are therefore in the range 6–30. Following feedback from the pilot, the wording of the anchors for the scores — *Not at all*, *Somewhat*, *A little*, *Very*, and *Extremely* — were rewritten for clarity to read: *Not at all*, *Slightly*, *Moderately*, *Very*, and *Extremely*.

This study directly manipulates the amount of adversarial dialogue in the stories, so the manipulation checks were aimed at quantifying this manipulation. Tallying the episodes of verbal conflict seemed a useful metric (see ESM 4 at Berks, 2026). However, these verbal behaviours are un-weighted, so our main manipulation check was perceived levels of conflict, using a question based on the format of the PSQI: “To what extent is the relationship between the two characters conflictual?”

Comprehension checks were also included to capture whether participants understood the story enough to give meaningful ratings. This involved three multichoice questions, each with three choices, testing recall of content and comprehension. Table 5 gives an example, and ESM 3 (see Berks, 2026) all the questions.

**Table 5:** Example of Multi Choice Comprehension Question

What does Shaggor want to take to the top of the pillar?	
o	His sword.
o	A rope.
o	A skull.

A preregistration (see Berks et al., 2020), and deidentified study data, analytics methods, and study materials may be found on the Open Science Framework at Berks et al. (2026a) for Experiment 1.

Furthermore, a preregistration (see Berks, 2022), and deidentified study data, analytics methods, and study materials may be found on the Open Science Framework at Berks et al. (2026b) for Experiment 2.

Additional material is contained in five sections of Electronic Supplementary Material (see Berks, 2026):

- ESM 1 Supplementary Data Analysis.
- ESM 2 Methodology in More Detail.
- ESM 3 Qualtrics Printout for Experiment 1.
- ESM 4 Conflict Behaviours.
- ESM 5 Qualtrics Printout for Experiment 2.

### Data Processing and Analysis

#### Analysis Software

R programming language was used, with additional packages from the Tidyverse (Wickham et al., 2019) and nlme (Pinheiro et al., 2013).

#### Data Quality Check

Comprehension checks were administered and reading time were measured.

## Results: Experiment 1

Table 6 gives descriptive statistics from Experiment 1.

**Table 6:** Descriptive Statistics for Experiment 1, High and Low Conflict Story Conditions

Measure	*M*	*SD*	SE	95% CI
PSQI				
High	18.89	4.16	0.61	[17.67, 20.12]
Low	19.28	4.07	0.59	[18.08, 20.47]
Entertainment				
High	3.02	1.05	0.15	[2.71, 3.33]
Low	2.98	1.01	0.15	[2.68, 3.28]
Conflict				
High	3.32	1.02	0.15	[3.02, 3.62]
Low	2.34	1.09	0.16	[2.02, 2.66]

Table 7 gives inferential statistics from Experiment 1, showing the manipulation of conflict led to a highly significant change in perceived conflict, with a large to very large effect size, whereas it had no significant effect (at a *p* < .05 level) on the measure of quality (total PSQI) or Entertainment, with small to very small effect sizes.

**Table 7:** Inferential Statistics for Experiment 1

Measure	Mean difference	95%CI	*t*-value	*p*-value	Cohen’s *d*
Perceived conflict	0.98	[-1.45, -.51]	*t*(46) = 4.21	<.001	0.61
PSQI Total	0.38	[-1.45, 2.22]	*t*(46) = 0.42	.68	0.06
Entertainment	0.04	[-0.51, 0.42]	*t*(46) = 0.18	.86	0.03

The loss of data, through losing Condition 7 (see Table 3) decreased the experiment’s power, and introduced potential bias by creating a correlation between conflict level and genre/story type — the low conflict stories were less likely to be romance stories, and the high conflict stories less likely to be fantasy stories. To address this, we created a multilevel model, with random intercepts for the three genres/story types, so controlling for the effect of genre/story type (see ESM 1 at Berks, 2026). The idea was both to reduce the noise in the PSQI ratings and thus increase power to detect an effect of conflict and get a sense of how much genre/story type might be affecting the results. For both the total PSQI and the Entertaining item, the intercept only model showed an intraclass correlation coefficient for the grouping variable (individuals) of .00, suggesting, somewhat surprisingly, that individual differences made no difference to either rating. The fixed effects for both the total PSQI and the Entertaining item suggested any effect of genre/story was insignificant.

While this study concerns the effect of conflict in stories in general, the effect of this conflict may vary across genre/story type, and so post hoc analyses of these effects are included in ESM 1 (Berks, 2026), showing no variation across genre.

## Discussion: Experiment 1

In Experiment 1, we increased the conflict in the dialogue by following recommendations in the literature (Patterson, 2013; Ware et al., 2012), which increased the perceived level of conflict between the characters. This increase in perceived level of conflict was both statistically significant and — using the criteria of a Cohen’s *d* of more than 0.8 (Cohen, 1988) — had a large to very large effect size. Yet participants did not rate the stories with conflictual dialogue as higher in quality or Entertainment than the stories with neutral dialogue. While this may reflect reality in this population, it also seemed plausible that this finding was a Type II error due to methodological flaws, particularly in either the measurement of the dependent variables, or having insufficient power, which might have affected both the *t*-tests and the multi-level models.

While the PSQI appeared a good option to study story quality and Entertainment, its validity has not been rigorously tested; and the PSQI total score and Entertainment may give insufficiently broad conceptualizations of story quality, and audience response. Moreover, we may have underestimated the sample size needed to detect an effect due to optimistic assumptions about the effect size. Any underestimate would be compounded when the exclusion criteria and imperfect randomization eliminated more participants than predicted (see ESM 2 at Berks, 2026).

These two issues led to the main design changes in Experiment 2.

### Hypotheses: Experiment 2

The first and second hypotheses was retained from Experiment 1.

*Hypothesis 3 (H3)*: high levels of dramatic conflict in story dialogue will result in higher enjoyment ratings than low levels of dramatic conflict.
*Hypothesis 4 (H4)*: high levels of dramatic conflict in story dialogue will result in lower boredom ratings than low levels of dramatic conflict.

Appreciation and suspense are other potential variables suggested by the literature but only included as exploratory analyses to maintain consistency with the preregistration.

## Method: Experiment 2

A second experiment with increased power was felt to be justified to confirm the findings of Experiment 1. Two other major changes were the use of a broader set of outcome measures and placing these measures at the completion of the stories, so participants would rate the stories as a whole.

### Participants

#### Recruitment

To avoid comprehension problems, participants were again recruited via Prolific with English fluency, and as an additional check, recruited from countries with English as the primary language (Government of the United Kingdom, 2022).

Two hundred and forty-one participants entered the study, after exclusions 194 remained (see ESM 2 at Berks, 2026).

#### Sample Size/Power Analysis

The study had a target sample size of 199 based on the power analysis. The same figures and calculations were used as for Experiment 1, except for the estimated Cohen’s *d*. Following the findings of Experiment 1, it was assumed that effect size would be small Cohen’s *d* of 0.2 (Cohen, 1988). Therefore, a minimum sample of 199 would be needed to detect a difference between the ‘low’ and ‘high’ conditions. Given a loss of data from exclusions, the recruitment target was set at 240.

### Procedure

The procedure was the same as Experiment 1 except participants read the whole story prior to answering the comprehension check, the story evaluation questions, and the manipulation check.

### Design

The design was once again a repeated measures experiment, with two levels of the independent variable, dramatic conflict; but this time with four main dependent variables: Story quality, Audience Response, Enjoyment, and boredom.

### Materials

#### Stories

The same three stories were used as for Experiment 1. However, they were rewritten so that the evaluation could be of the whole story (materials and measures are in ESM 5). Conflict was generated, and conflict behaviours tallied, in the same manners as for Experiment 1 (see ESM 4 at Berks, 2026).

#### Measures

We implemented four measures.

1. Story quality was measured by a single question on a 5-point rating scale, asking participants to complete the sentence: “This story was … very bad/bad/neither good nor bad/good/very good.” Asking people to rate story goodness is a common way of measuring story quality in the literature (Kemper et al., 1990; McCabe & Peterson, 1984; Pratt & Robins, 1991; Stein & Policastro, 1984) and was chosen to try to capture a wider conception of story quality than the PSQI.
2. The Audience Response Scale (ARS) (Oliver & Bartsch, 2010) was chosen for Experiment 2, because it offers a broader conception of audience response than Entertainment, gives an overall rating for Audience Response, contains multiple dimensions (comprising evenly divided subscales for *Enjoyment*, *Appreciation*, *Suspense*, and *Lasting impression*), and has been shown to have good psychometric properties (Angulo-Brunet & Soto-Sanfiel, 2020; Bartsch & Hartmann, 2017; Johnson et al., 2015; Johnson & Rosenbaum, 2015; Oliver & Bartsch, 2010; Price, 2017; Schols, 2018). This 12-item scale measures a wide range of positive audience cognitive/emotional responses to media. Participants rate their agreement on a 7-point Likert scale (Strongly disagree/Disagree/Somewhat disagree/Neither agree nor disagree/Somewhat agree/Agree/Strongly agree) with a series of statements. Total scores for the ARS are in the range 12–84. The ARS is evenly divided into four sub-scales (*Enjoyment*, *Appreciation*, *Lasting impression*, *Suspense*).
3. Participants judged boredom on a 7-point Likert scale, rating their agreement (Strongly disagree/Somewhat disagree/Neither agree nor disagree/Somewhat agree/Agree/Strongly agree) with: “This story was boring.” This was included based on evidence (Schindler et al., 2017), that negative emotions in response to media, including boredom, appear to form a separate construct.
4. Participants judged conflict on a 7-point Likert scale, which asked participants to rate their agreement (Strongly disagree/Somewhat disagree/Neither agree nor disagree/Somewhat agree/Agree/Strongly agree) with: “The characters in this story were in conflict.” This was a change in prompt from Experiment 1 to more closely followed the style of the ARS. The comprehension checks were the same format as Experiment 1.

## Results: Experiment 2

Table 8 gives descriptive statistics for Experiment 2.

**Table 8:** Experiment 2 Descriptive Statistics

Measure	*M*	*SD*	SE	95% CI
Quality				
High	3.31	0.88	0.06	[3.18, 3.43]
Low	3.38	0.92	0.07	[3.25, 3.51]
ARS Total				
High	40.49	14.3	1.03	[38.46, 42.52]
Low	40.88	15.44	1.11	[38.7, 43.07]
Enjoyment				
High	13.51	4.5	0.32	[12.87, 14.15]
Low	13.71	4.73	0.34	[13.04, 14.38]
Boredom				
High	3.63	1.7	0.12	[3.39, 3.88]
Low	3.6	1.7	0.12	[3.36, 3.84]
Conflict				
High	4.69	1.4	0.1	[4.49, 4.89]
Low	3.88	1.57	0.11	[3.66, 4.10]

Table 9 gives inferential statistics, indicating that while manipulating the independent variable, *Conflict*, produces a lower effect size than Experiment 1, this change remains significant. Other dependent variables show similar effects to Experiment 1 — no significant effects (at a *p* < .05 level) for *Quality*, *Audience Reaction*, *Enjoyment*, or *Boredom*.

**Table 9:** Experiment 2 Inferential Statistics

Measure	Mean difference	95% CI	*t*-value	*p*-value	Cohen’s *d*
Quality	0.07	[-.07, 0.21]	*t*(193) = 1.01	.32	0.07
ARS Total	0.39	[-1.76, 2.54]	*t*(193) = 0.36	.72	0.03
Enjoyment	0.2	[-.53, 0.93]	*t*(193) = 0.54	.59	0.04
Boredom	0.03	[-.32, 0.25]	*t*(193) = 0.21	.83	0.02
Conflict	0.81	[-1.09,-0.53]	*t*(193) = 5.74	<.01	0.41

A multi-level model with fixed slopes (see ESM 1 at Berks, 2026) for *Quality*, *ARS Total*, *Enjoyment*, and *Boredom*, did not show conflict level having a significant effect (at a *p* < .05 level). In the exploratory analyses, descriptive and inferential statistics (Table 10 and Table 11) of subcategories of the ARS, including Appreciation and Suspense were calculated, showing no significant effects (at a *p* < .05 level).

**Table 10:** Experiment 2 Exploratory Analyses: Descriptive Statistics

Measure	*M*	*SD*	SE	95% CI
Appreciation				
High	9.68	4.13	0.3	[9.09, 10.26]
Low	9.85	4.47	0.32	[9.21, 10.48]
Memorable				
High	7.6	3.69	0.26	[7.08, 8.12]
Low	7.78	4.03	0.29	[7.21, 8.35]
Suspense				
High	9.71	4.29	0.31	[9.10, 10.31]
Low	9.54	4.35	0.31	[8.93, 10.16]

**Table 11:** Experiment 2 Exploratory Analyses: Inferential Statistics

Measure	Mean difference	95% CI	*t*-value	*p*-value	Cohen’s *d*
Appreciation	0.17	[-0.49, 0.83]	*t*(193) = 0.51	.61	0.04
Memorable	0.19	[-0.3, 0.67]	*t*(193) = 0.75	.45	0.05
Suspense	-0.16	[-0.87, 0.54]	*t*(193) = 0.46	.65	0.03

Analyses of the interaction between conflict levels and genre/story type are included in ESM 1, showing instances of effects varying across genre/story type.

## Overall Discussion

These experiments investigated whether dramatic conflict, in the form of adversarial dialogue, improved audience ratings of story quality, increased positive emotional/cognitive reactions to a story, or decreased negative reactions.

Making changes to the dialogue, as recommended by the literature, in order to increase dramatic conflict (Patterson, 2013; Stein, 2014) increased the perceived level of conflict in both experiments. The change was statistically significant and had a large to very large effect size (Cohen, 1988). Despite the successful manipulation of conflict, in neither experiment did participants rate the stories with conflictual dialogue more positively over a range of dependent variables. Ostensibly, this is in contrast to experiments showing that people have greater liking for stories with conflict (Brewer & Lichtenstein, 1981; Diener & Woody, 1981; Jose, 1988), and expert opinion that adversarial dialogue is more entertaining (Axelrod, 2013; Bell, 2014; Frey, 2010; Hough, 2015; Stein, 1995).

There are several possible explanations for these findings.

One explanation is that there was no measurable effect because increasing the conflict level in dialogue has no positive effect on audience reaction. Therefore, these results provide no support for the dramatic theory of conflict. There is a parallel in the literature on violence in the media, which has largely failed to show a rise in audience enjoyment with higher levels of violence (Weaver, 2011). However, this lack of positive effect is what would have been predicted by the traditional theory of conflict, which posits that dramatic conflict has a positive effect when it is a consequence of structural conflict. The traditional theory also offers an explanation for the positive effect of conflict in previous experiments which manipulated story conflict (Brewer & Lichtenstein, 1981; Diener & Woody, 1981; Jose, 1988), as it appears likely these were manipulating structural conflict or a combination of structural and dramatic conflict. This null result is also compatible with the psychological theory, which does not have dramatic conflict as a key variable.

Another explanation for the results is that the effect of adversarial conflict was obscured by methodological issues. Potential issues include the strength or dynamics of the manipulation, the studies’ power, and measurement problems.

A stronger manipulation of conflict may have produced a significant response in the dependent variables. Against this, are the response in the manipulation check, and that nothing in the literature suggests a response threshold. More plausibly, conflict that built to a high point and then resolved might have produced more of an effect than the ‘static’ conflict created in these experiments (Egri, 2009; Freytag, 1896). However, characters are expected to act believably (Bates, 1994), and characters engaging in a high level of conflict, either static or building to a peak, which is unrelated to the structural conflict may violate audience expectations.

The study may have been underpowered to detect a very small effect. Despite Experiment 2 having a large sample, it did not achieve the target sample size.

Finally, story-level measures may not be sensitive enough to detect people’s response to dramatic conflict in dialogue. In both studies, participants were asked to rate their reactions to the whole story on the assumption that improvements to dialogue in the dialogue heavy story would translate to better ratings of the story as a whole. If instead, participants were asked to rate a passage of dialogue as a passage, this tighter focus might have allowed detection of meaningful change.

Beyond these limitations, these findings may not generalize. The research tested one form of dramatic conflict, using three different stimuli, in one specific medium (online written stories), consequently, these results only permit strong inferences about the specific stories and medium studied, and cannot necessarily be generalized to a wider “population” of stories and media (Judd et al., 2012; Yarkoni, 2022).

An additional methodological issue, which is unlikely to have affected the result, but could affect its interpretation, is the extent that dramatic conflict was isolated from structural conflict. As there was no check for this isolation in the study design, caution is warranted in interpreting these results as pertaining solely to the effects of dramatic conflict.

The importance of these experiments is in providing practical guidance to writers and helping fill a gap in the current empirical and theoretical literature on the importance of conflict in stories.

While this research is in the form of a psychology experiment, in which a stimulus is manipulated, and people’s responses recorded, it is based on a practical problem. If a passage of dialogue in a story is flat, is it enough for the writer to merely increase the conflict? The results suggest caution against simply adding dramatic conflict to dialogue in the hope of improving story quality and audience reaction. How then should writers approach conflict in dialogue? Firstly, the writer could give any conflict an outcome (something deliberately avoided in these experiments). Previous research into the psychological theory of conflict, strongly suggests that writing dialogue in which a liked protagonist wins a verbal contest with an unliked antagonist will provide enjoyment to an audience, while the reverse outcome will lead to appreciation. This audience response will happen whether or not the adversarial dialogue is strongly linked to structural conflict. Secondly, but more speculatively, there is nothing in these findings which would prevent a writer following the expert opinion in the form of the traditional theory of conflict, where adversarial dialogue springs from the protagonists’ story goals, and obstacles to those goals.

Indeed, the lack of effect on an audience of isolated dramatic conflict, if supported by other research, would turn our attention away from the dramatic theory of conflict to the traditional and psychological theories. Here understanding the effect of dramatic conflict remains important but is only one piece of the puzzle.

This study suggests that future research into conflict could include measuring story quality and audience reaction to different manipulations: manipulating structural conflict while keeping a dramatic scene unchanged; manipulating structural conflict without any dramatic conflict; or manipulating dramatic conflict in a story in which there is a clear valence of character and/or story resolution. Alternatively, research might have a narrower focus, asking participants to rate just a passage of dialogue in which the dramatic conflict has been manipulated. Moving beyond this, the effect of internal vs external conflict, or static conflict versus conflict that builds to a peak could be investigated.

In conclusion, this study examined an under researched part of a broader literature on conflict in stories: whether isolated dramatic conflict, in the form of adversarial dialogue, improves story quality and the audience reaction to the story. No evidence was found supporting this idea. In the absence of similar research, one should not over interpret these results, yet they suggest conflict theories need further experimental testing.

## Supplementary Materials

**Table:** 

Type of supplementary materials	Availability/Access
Data	
Deidentified study data - Experiment 1.	Berks et al. (2026a)
Deidentified study data - Experiment 2.	Berks et al. (2026b)
Code	
R code - Experiment 1.	Berks et al. (2026a)
R code - Experiment 2.	Berks et al. (2026b)
Material	
Dialogue and question prompts - Experiment 1.	Berks et al. (2026a)
Question prompts and survey - Experiment 2.	Berks et al. (2026b)
Study/Analysis preregistration	
Experiment 1 - Preregistration.	Berks et al. (2020)
Experiment 2 - Preregistration.	Berks (2022)
Other	
Codebook - Experiment 1.	Berks et al. (2026a)
Codebook - Experiment 2.	Berks et al. (2026b)
Electronic Supplementary Material (1–5).	Berks, 2026

## Data Availability

A preregistration (see Berks et al., 2020), and deidentified study data, analytics methods, and study materials may be found on the Open Science Framework at Berks et al. (2026a) for Experiment 1. Furthermore, a preregistration (see Berks, 2022), and deidentified study data, analytics methods, and study materials may be found on the Open Science Framework at Berks et al. (2026b) for Experiment 2. Additional material is contained in five sections of Electronic Supplementary Material (see Berks, 2026): ESM 1 Supplementary Data Analysis, ESM 2 Methodology in More Detail, ESM 3 Qualtrics Printout for Experiment 1, ESM 4 Conflict Behaviours, ESM 5 Qualtrics Printout for Experiment 2.
